# Profiling the Genome-Wide Landscape of Short Tandem Repeats by Long-Read Sequencing

**DOI:** 10.3389/fgene.2022.810595

**Published:** 2022-05-05

**Authors:** Zhenhua Liu, Guihu Zhao, Yuhui Xiao, Sheng Zeng, Yanchun Yuan, Xun Zhou, Zhenghuan Fang, Runcheng He, Bin Li, Yuwen Zhao, Hongxu Pan, Yige Wang, Guoliang Yu, I-Feng Peng, Depeng Wang, Qingtuan Meng, Qian Xu, Qiying Sun, Xinxiang Yan, Lu Shen, Hong Jiang, Kun Xia, Junling Wang, Jifeng Guo, Fan Liang, Jinchen Li, Beisha Tang

**Affiliations:** ^1^ Department of Neurology, Xiangya Hospital, Central South University, Changsha, China; ^2^ National Clinical Research Center for Geriatric Disorders, Xiangya Hospital, Central South University, Changsha, China; ^3^ GrandOmics Biosciences, Beijing, China; ^4^ Department of Geriatrics, The Second Xiangya Hospital, Central South University, Changsha, China; ^5^ Multi-Omics Research Center for Brain Disorders, The First Affiliated Hospital of University of South China, Hengyang, China; ^6^ Department of Geriatrics, Xiangya Hospital, Central South University, Changsha, China; ^7^ Key Laboratory of Hunan Province in Neurodegenerative Disorders, Central South University, Changsha, China; ^8^ Centre for Medical Genetics and Hunan Key Laboratory of Medical Genetics, School of Life Sciences, Central South University, Changsha, China

**Keywords:** short tandem repeats, long-read sequencing, highly variable STRs, TRcards, database, brain tissue, synaptic function

## Abstract

**Background:** Short tandem repeats (STRs) are highly variable elements that play a pivotal role in multiple genetic diseases and the regulation of gene expression. Long-read sequencing (LRS) offers a potential solution to genome-wide STR analysis. However, characterizing STRs in human genomes using LRS on a large population scale has not been reported.

**Methods:** We conducted the large LRS-based STR analysis in 193 unrelated samples of the Chinese population and performed genome-wide profiling of STR variation in the human genome. The repeat dynamic index (RDI) was introduced to evaluate the variability of STR. We sourced the expression data from the Genotype-Tissue Expression to explore the tissue specificity of highly variable STRs related genes across tissues. Enrichment analyses were also conducted to identify potential functional roles of the high variable STRs.

**Results:** This study reports the large-scale analysis of human STR variation by LRS and offers a reference STR database based on the LRS dataset. We found that the disease-associated STRs (dSTRs) and STRs associated with the expression of nearby genes (eSTRs) were highly variable in the general population. Moreover, tissue-specific expression analysis showed that those highly variable STRs related genes presented the highest expression level in brain tissues, and enrichment pathways analysis found those STRs are involved in synaptic function-related pathways.

**Conclusion:** Our study profiled the genome-wide landscape of STR using LRS and highlighted the highly variable STRs in the human genome, which provide a valuable resource for studying the role of STRs in human disease and complex traits.

## Introduction

Short tandem repeats (STRs) are abundant repetitive elements comprised of recurring DNA motifs of two–six bases. Due to their repetitive nature, STRs have the highest mutational rate in the genome and are typically polymorphic. They are often used in forensics and population genetics and are also the underlying cause of many genetic diseases (Gymrek 2017; Hannan 2018).

STR expansions in the coding or non-coding regions are linked to more than 50 known disorders (Depienne and Mandel, 2021). Many of these conditions affect the nervous system. Well-known examples of STR expansion diseases in protein-coding regions are the “polyglutamine” (PolyQ) diseases (e.g., Huntington disease and Spinocerebellar ataxia), caused by variable stretches of the repeated trinucleotide CAG. Non-coding repeat expansions are even more diverse and can occur in either the 5′ UTRs, introns, or 3′ UTRs of genes. Their impact strongly depends on the type, length, and location of the repeat motif within genes. Examples of these repeat disorders include Fragile X syndrome (FXS) caused by CGG repeats and Myotonic dystrophy (DM1) caused by CTG repeats (Tang et al., 2017; Trost et al., 2020; Depienne and Mandel, 2021).

Recently, more than 28,000 eSTRs in 17 tissues were identified to play a role in gene regulation by leveraging deep whole-genome sequencing (WGS) and gene expression data collected by the Genotype-Tissue Expression Project (GTEx), STRs for which the number of repeats was associated with the expression of nearby genes, termed expression STRs (eSTRs). Then, eSTRs were ranked with a statistical fine-mapping framework to prioritize potentially causal eSTRs and 5% of which were referred to as fine-mapped eSTRs (FM-eSTRs) (Fotsing et al., 2019). It is becoming increasingly clear that STRs across the genome are likely to have widespread contributions to complex polygenic traits. In these cases, smaller expansions or contractions may subtly increase or decrease the risk for a trait and work together to modulate an individual’s disease risk (Gymrek et al., 2016; Fotsing et al., 2019; Jakubosky et al., 2020).

Genome-wide surveys of STRs in individual genomes have become feasible due to the development of high-throughput sequencing technologies. Most studies used whole-genome sequence data based on short-read sequencing (SRS) to genotype STRs (Willems et al., 2014; Tang et al., 2017; Mousavi et al., 2019; Trost et al., 2020; Mitra et al., 2021). However, the intrinsic limitations of SRS prevent the comprehensive characterization of all STRs or the discovery of novel disease-relevant repeat expansions, which are longer than read length (Gymrek, 2017; Liu et al., 2020).

Long-read sequencing (LRS) technologies offer a good solution to genome-wide STR analysis. Current LRS technologies, such as Pacific Biosciences sequencing and Oxford Nanopore Technologies (ONT) sequencing, have achieved reads longer than 10 kb on average, which have a high chance to cover whole tandem repeats, including flanking unique sequences (Pollard et al., 2018; Midha et al., 2019; Amarasinghe et al., 2020; Logsdon et al., 2020). LRS has recently been applied to genotype long and complex repeats, such as the *C9orf72* GGGGCC expansion implicated in frontotemporal lobar degeneration and a complex pentamer repeat in *SAMD12* implicated in myoclonus epilepsy (Zeng et al., 2019; Mitsuhashi and Matsumoto, 2020; DeJesus-Hernandez et al., 2021). More human diseases caused by STR expansions have also been reported in recently published studies with the utilization of LRS (Sone et al., 2019; Tian et al., 2019; Zeng et al., 2019; Deng et al., 2020).

The normal ranges of different STRs may vary significantly in the general population. Thus, the knowledge of the normal repeat ranges of STRs is critically important to determine that the pathogenicity of observed repeats in known STRs or to discover novel disease-relevant repeat expansions (Liu et al., 2020). To the best of our knowledge, although there exist studies on detecting and characterizing STRs in human genomes using LRS on select small datasets, analysis at scale has not been reported (Liu et al., 2020).

Herein, we conducted a large-scale analysis of human STR variation by LRS in the Chinese population and developed a reference STR database, named TRcards, with 193 of the LRS dataset. Besides, we performed genome-wide profiling of STR variation in the human genome with LRS data, evaluated the variability of STR and characterized the highly variable STRs.

## Materials and Methods

### Participants

A set of 193 unrelated Chinese was included in our study for ONT sequencing. Among all the individuals, 102 (52.85%) were males and 91 (47.15%) were females. The ages ranged from 26 to 85 years, with a median age of 50 years. This study was approved by the Ethics Committee of Xiangya Hospital, Central South University. All participants gave informed consent.

### Long-Read Whole-Genome Sequencing

DNA samples sequenced in this study were isolated from whole blood. DNA samples of individuals were sequenced using a PromethION sequencer (Oxford Nanopore Technologies). Library preparation was carried out using a 1D Genomic DNA ligation kit (SQKLSK109) according to the manufacturer’s protocol. For each individual, one PRO-002 (R9.4.1) flow cell was used. PromethION data base-calling was performed using guppy v.3.3.0 (Oxford Nanopore Technologies), and only pass reads (Qscore ≥7) were used for subsequent analysis (Sun et al., 2020).

Sample LNT00178 was also sequenced with the Pacibio Sequel II platform. High molecular weight (HMW) DNA was extracted, and HiFi libraries were constructed using the SMRTbell Express Template Prep Kit v2 and SMRTbell Enzyme Clean Up Kit (PacBio) (Du et al., 2021). Size selection was performed with SageELF and 15 kb fragments were chosen for sequencing with the Sequel II platform using 30 h movies. Then, the resulting raw subreads were converted to circular consensus sequencing (CCS) reads using the CCS v4.2 algorithm with–minPasses 3 –minPredictedAccuracy 0.99. Furthermore, HG002 with ONT and the corresponding PacBio CCS data were downloaded from https://ftp-trace.ncbi.nlm.nih.gov/ReferenceSamples/giab/data/AshkenazimTrio/HG002_NA24385_son/UCSC_Ultralong_OxfordNanopore_Promethion/ and https://ftp-trace.ncbi.nlm.nih.gov/ReferenceSamples/giab/data/AshkenazimTrio/HG002_NA24385_son/PacBio_SequelII_CCS_11kb/, respectively. The ∼15X CCS data and ONT data were randomly chosen using samtools views and were used for the following comparison.

### STR Detection

Based on the RepeatMasker result from UCSC Genome Browser, we prepared a gene-associated STR list spanning all GENCODE V19 genes. Genes upstream and downstream of the 10 kb region and the STR repeat unit ranged in length from 3 to 6 bp. The pass reads from PromethION were aligned to the reference genome hg19 using ngmlr v.0.2.7 with -x ONT (Sedlazeck et al., 2018).

For each repeat, the repeat count of each read that aligned with the STR locus was detected using RepeatHMM v2.0.3 without the peak calling step (Liu et al., 2017). RepeatHMM used a template with perfect repeats to correct sequencing errors, and then a repeat count of each read was given using the HMM model. The repeat counts could contain other motifs similar to the target motif. The repeat counts are ranked by decreasing repeat size, and the repeat size located at the top 25% was defined as the individual’s estimated repeat count (ERC). After that, repeat counts of all STRs whose repeat counts are successfully detected are combined in a single output file. Then, we merged the STRs detected from all the samples for each STR locus and constructed a merged STR dataset. A minimum of 8x coverage for STR loci is required to infer the repeat size. If the coverage is less than 8x on the predefined STR loci in samples, the repeat count was discarded.

### PacBio HiFi Comparison

In this study, the HiFi CCS reads of HG002 and individual S004860 were aligned to the reference genome Hg19 using minimap2 with -ax asm20 -t 40 --MD -Y -L (Li 2018). The corresponding ONT reads were aligned using ngmlr v.0.2.7 with -x ONT as a previously described method in this study. Then, the repeat counts of each STR were calculated using RepeatHMM v2.0.3 with-SeqTech Pacbio and-SeqTech Nanopore, respectively. The Pearson correlation coefficient was used to assess the correlation between these two results.

### STR Categories

The full catalog of STR variations detected in our dataset is publicly available at TRcards (http://www.genemed.tech/trcards/home). We defined our STR categories with respect to their motif size, genomic regions, and repeat units. For the repeat unit, the reverse complement sequences and base order were considered (e.g., the pattern of CAG and its derived sequences, including GTC, GCA, AGC, CTG, GCT, and TGC). More than 50 disease-related STR (dSTR) loci are reported to cause disorders (Depienne and Mandel, 2021). dSTRs were subdivided into different classes based on the repeat unit. The classes are repeat unit CAG, repeat unit CCG, and repeat unit TTTTA (Ishiura and Tsuji, 2020; Mitsuhashi et al., 2021).

A population-scale analysis of the STR variation database WebSTR was developed by Richard Yanicky and Melissa Gymrek based on 1,000 Genomes samples (Mallick et al., 2016; Gymrek et al., 2017). An overlap STR catalog between our database and WebSTR was defined.

Expression STRs (eSTRs) and the top fine-mapped eSTRs (FM-eSTRs) catalog were reported by Fotsing et al. (2019). An overlap STR catalog between our database and eSTR was defined.

### Scoring the Variability of STR

We introduce the repeat dynamic index (RDI) to score a specific STR variability. After sorting the 193 repeat counts for a repeat locus, we obtain repeat counts between the maximum fifth percentile value and 95th percentile value to represent a robust normal repeat range so that the minimum and maximum outliers are excluded. Then, RDI is defined as the Standard Deviation of the normal repeat range in our dataset after removing the STR repeat counts above the fifth percentile or low 95th percentile. RDI is calculated using $\sqrt{\sum {\left(R-\bar{Ri}\right)}^{2}/N}$ . In this formula, *N* is the number of samples after removing samples with the minimum and maximum outliers, $\bar{Ri}$ is the mean of the *N* repeat counts, and *R* is a repeat count in a specific rank. RDI models the relationship between the median reference repeat size and the variability of STR. We ranked STRs by their RDI score and then transferred them into the normalized RDI score in our STR catalog. We referred to the STR with normalized RDI score at 0–0.2, 0.2–0.4, 0.4–0.6, 0.6–0.8, and 0.8–1.0 as very lowly variable (vlSTR), lowly variable STR (lSTR), moderately variable STR (mSTR), highly variable STR (hSTR), and very highly variable STR (vhSTR), respectively.

### Characterizing the Expression Pattern of vhSTRs and hSTR

To explore the tissue specificity of vhSTR and hSTR related genes across tissues, we sourced the expression data from the Genotype-Tissue Expression (GTEx) database (Consortium et al., 2017). The average expression level of each gene in each tissue was calculated. Because GTEx experiments were conducted at a set read depth for all tissue samples, cross-tissue comparisons with these tissues could be biased (Feiglin et al., 2017). To address this potential bias, we substituted the expression values of each gene with their rank in the sample. Normalization was performed separately for each tissue using the R package (Li et al., 2021). The rank of the normalized gene expression values was defined as the normalized tissue expression value. We then used normalized mean expression values to assess the expression profile in different tissues.

### Enrichment Analysis of vhSTRs and hSTR

To identify potential functional roles of the high variable STRs, Gene Ontology (GO) analysis of the biological process (BP), cellular component (CC), and Molecular Function (MF) levels and Kyoto Encyclopedia of Genes and Genomes (KEGG) pathway analysis were performed using the cluster Profiler package. An adjusted *p*-value < 0.01 was considered statistically significant, and the visualization of results was performed with the GO plot package. The *p*-value was calculated with Fisher’s exact test, and multiple testing of *p*-values was corrected by the Benjamini–Hochberg method.

### TRcards Database Construction and Interface

TRcards was developed using JavaScript, PHP, and Perl using a Linux platform on a Nginx web server. A front and back separation model was used. The front end was based on vue and used the UI Toolkit element, which supports all modern browsers across platforms, including Microsoft Edge, Safari, FireFox, and Google Chrome. The back end was based on Laravel, a PHP web framework. The front and back separation model has many advantages, including simplicity of control, modularity, and expandability. TRcards is compatible with all major browser environments and different operating systems, including Windows, Linux, and Mac. The data were stored in a MySQL database.

### Statistic

The statistical tests used were described throughout the article and in the figures. We performed FDR correction for multiple comparisons. The enrichment analysis was conducted by Fisher’s exact test. The Benjamini–Hochberg corrected *p*-value was used for multiple test analysis. Pearson’s correlation coefficient was estimated for correlation analysis. All statistical tests were performed in the R package.

## Result

### Participants and Long-Read Sequencing Data

We performed whole-genome LRS for 193 unrelated Chinese using Oxford Nanopore PromethION as previously described (Sun et al., 2020). The detailed demographic information of the dataset is listed in Supplementary Table S1. An average of 53.95 Giga bases cleaned sequences were generated in those 193 individuals with an average of read length N50 up to 25.49 kb. Then, we mapped all cleaned reads to the human reference genome Hg19 and obtained an average depth of approximately 17.5X (range: 12.0X-45.7X). Base mapping rate for individuals varied from 79.48% to 99.08%, with an average of 95.31%, and the mean sequencing error rate was 11.53% (range: 8.32%–15.27%) (Supplementary Table S1, Supplementary Figure S1). Overall, our long-read sequencing data generated here showed high-coverage and high-quality, similar to two LRS-based structure variation studies in a population scale (Beyter et al., 2021; Wu et al., 2021).

### STR Detection and Validation

In total, 106,788 STRs coordinated with the Hg19 human reference genome were included in our dataset (Supplementary Table S2). About 70% of these loci are tri-nucleotide and tetra-nucleotide STRs and the remaining loci are penta-nucleotide and hexa-nucleotide STRs. Approximately 3,592 loci overlap coding region, 30,493 loci in the intronic proximal region (defined as the location within 1 kb from the nearby gene), 50,400 loci in the distal region of the intron (defined as a location more than 1 kb away from the nearby gene), 3,230 loci in the untranslated region (UTR), 10,351 loci in the upstream region, and 8,722 loci in the downstream region. The 20 most common STR units in our STR catalog were also listed (Supplementary Figure S2). All the STR catalogs and subsets were listed (Supplementary Tables S2, S3, S4). We further examined the reads coverage with different STR categories according to different motif sizes, genomic regions, and repeat units. As shown in Supplementary Figure S2, the sequencing coverage is relatively high, and the proportion of sequencing depth greater than eight layers exceeds 90% in all STR categories, which demonstrated our LRS data with high coverage for genotyping STRs.

To understand the accuracy of estimated repeat counts, we performed validation of estimated repeat counts using PacBio high-fidelity (HiFi) sequencing. We compared STR repeat counts with the LRS data from the individuals who were sequenced at the ONT and PacBio HiFi sequencing platform. PacBio HiFi sequence reads were both long and highly accurate (greater than 99%) and were computationally analyzed by RepeatHMM (Liu et al., 2017; Wenger et al., 2019) (Supplementary Table S1). After applying stringent recommended quality filters, STRs called from both platforms showed extremely high concordance with a strong correlation between estimated repeat counts reported by each (Pearson *r* = 0.8995; *p* < 2.2e-16) (Supplementary Figure S3). Our analysis proved that our ONT data could robustly genotype STRs and PacBio HiFi.

To further validate our results, we used capillary electrophoresis to genotype a subset of known disease-associated STR (dSTR) loci (Depienne and Mandel, 2021). Consequently, we compared the concordance between repeat sizes inferred by ONT and those obtained using capillary electrophoresis, the conventional standard for sizing STR loci. The repeat count estimated from the ONT was largely consistent with the capillary electrophoresis (Pearson *r* > 0.7; *p* < 2.2e-16) (Supplementary Figure S4).

Collectively, these validation results suggest that the repeat counts inferred by ONT are relatively accurate and demonstrate that ONT can deduce population-scale patterns of human STR variations.

### Genome-wide STR Profiling

**FIGURE 1:**
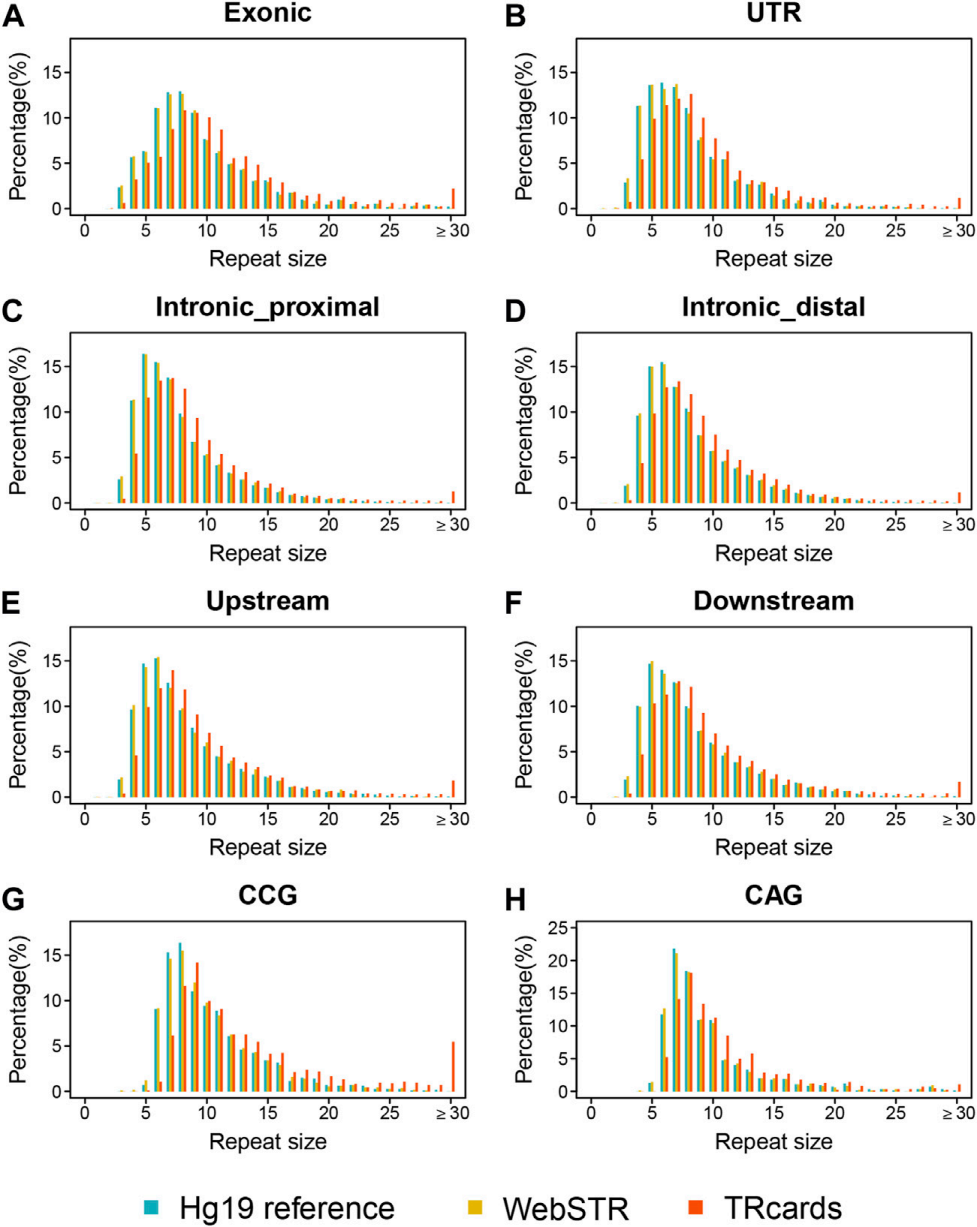
Genome-wide profiling of STR compared with published databases. **(A–F)** Distribution of repeat sizes in TRcards, Hg19 reference, and WebSTR database by stratifying STR according to genomic features, including the exonic region **(A)**, the untranslated region (UTR) **(B)**, the intronic proximal region (defined as the location within 1 kb from the nearby gene) **(C)**, the distal region of the intron (defined as a location more than 1 kb away from the nearby gene), the upstream region **(E)**, and the downstream region **(F)**. **(G–H)** Distribution of repeat sizes for CCG unit **(G)** and CAG units **(H)** in TRcards, WebSTR, and Hg19 reference database. Repeat unit CCG and repeat unit CAG are common STR repeat units reported to cause genetic diseases. In all panels, blue = Hg 19 reference; yellow = WebSTR; red = TRcards (our dataset). The *x*-axis shows the repeat size (repeat sizes above 30 are combined together). The *y*-axis shows the percentage of total STR loci.

We merged the repeat counts for all available STRs in the human reference genome with 193 available ONT long-read sequencing datasets to profile genome-wide STR variations. Several studies have used SRS data to genotype STRs on a population scale (Willems et al., 2014; Tang et al., 2017; Mousavi et al., 2019; Trost et al., 2020). Encouraged by the accuracy and scalability of our LRS dataset, here, we compared the referenced repeat sizes in our dataset to the STR sets provided by the Hg19 reference and WebSTR database (Willems et al., 2014; Mallick et al., 2016; Gymrek et al., 2017). We integrated those overlap STR loci between our dataset, Hg19 reference, and WebSTR database to define as an overlap STR catalog and then classified those overlap STR loci into different STR categories according to genomic features and repeat units. We found that the repeat size distribution of different STR categories (including different genomic features and repeat units) in our dataset is very similar to the published databases, but, in general, repeat sizes of our LRS dataset are larger than the Hg19 reference and WebSTR database with SRS data. Compared with the Hg19 reference and WebSTR database, the proportion of above 30 repeat sizes, especially in high GC content repeat loci (such as CCG unit), was significantly higher in our LRS data (Figure 1). Although SRS data were used to genotype STRs, it encountered difficulties reconstructing the expansion and underestimated the repeat sizes because of sequencing length, misalignment, and GC bias. In contrast, LRS can span the entire expansion and potentially help increase both the precision and the range of detectable variants.

**FIGURE 2:**
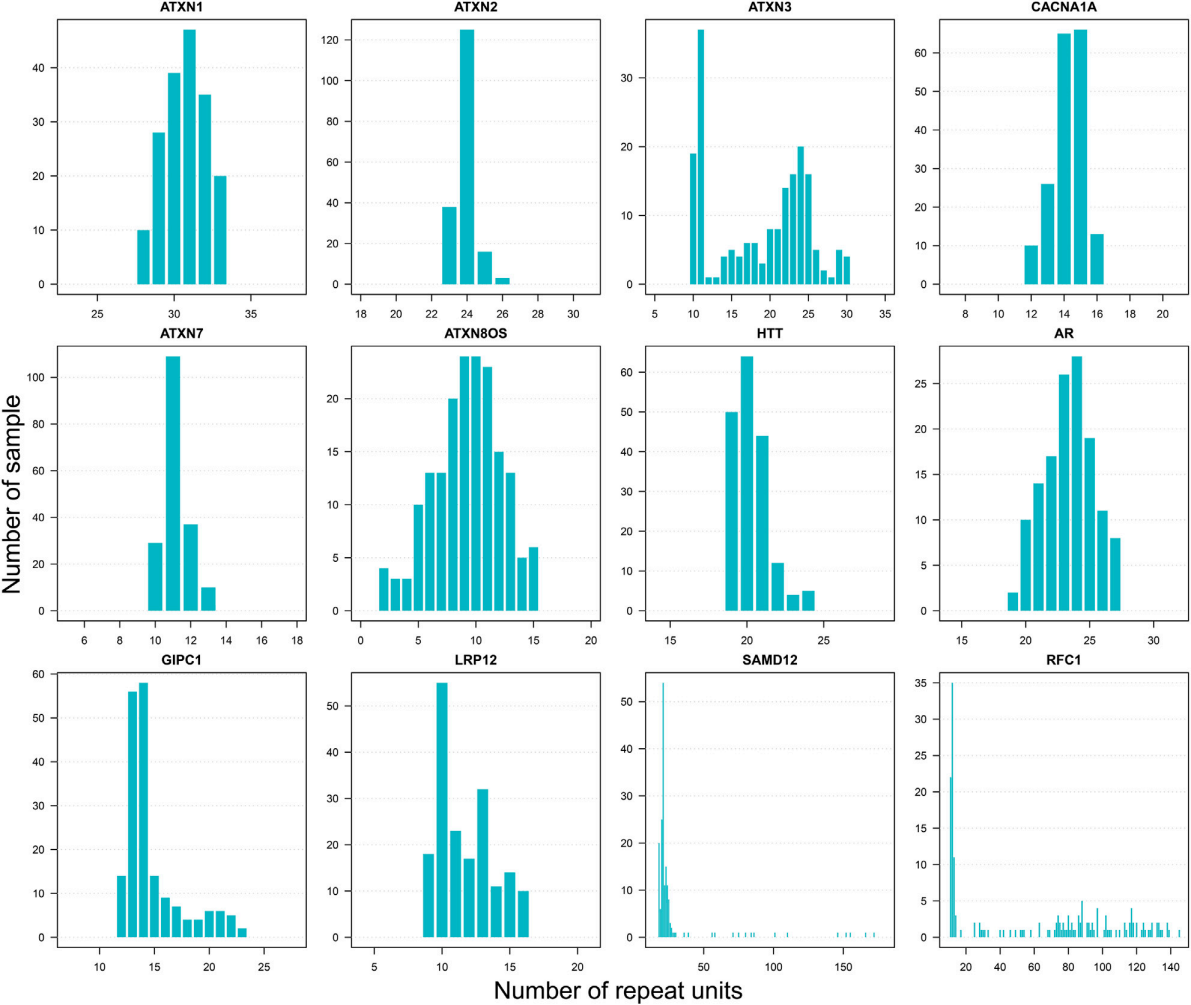
Distribution of repeat sizes for disease-associated STR loci. Selected well-studied disease-associated STR loci. Repeat unit CAG includes ATXN1 (Spinocerebellar Ataxia Type 1), ATXN2 (Spinocerebellar Ataxia Type 2), ATXN3 (Spinocerebellar Ataxia Type 3), CACNA1A (Spinocerebellar Ataxia Type 6), ATXN7 (Spinocerebellar Ataxia Type 7), ATXN8OS (Spinocerebellar Ataxia Type 8), HTT (Huntington’s disease), and AR (Spinal and Bulbar Muscular Atrophy). Repeat unit CCG includes GIPC1 (oculopharyngodistal myopathy) and LRP12 (oculopharyngodistal myopathy). Repeat unit TAAAA includes SAMD12 (familial cortical myoclonic tremor with epilepsy). Repeat unit AAAAG includes RFC1 (cerebellar ataxia, neuropathy, and vestibular areflexia syndrome).

The normal ranges of different STRs may vary significantly. Because repeat sizes can be accurately obtained from LRS data, STR analyses based on LRS data on a population scale could better estimate the referenced normal range of STR. Herein, we presented the distribution of repeat sizes at known disease-associated STR (dSTR) loci with our LRS dataset. After sorting the 193 repeat counts for a specific dSTR locus, we obtained repeat counts between the minimum value and top fifth percentile value to represent a robust normal repeat range so that maximum outliers are excluded. We found that the distribution of repeat sizes displayed either single peak or multiple peaks in dSTR loci, reflecting genetic variability in the general population. Evaluation of well-studied dSTR loci (e.g., *ATXN1*, *ATXN2*, *ATXN3*, and *HTT*) showed that the repeat ranges inferred by our LRS data provided good estimation to repeat ranges reported in the literature from population-scale studies. Besides, other dSTR loci, which have not been well-characterized in literature (e.g., *GIPC1* and *LRP12*), were also evaluated in our dataset, and we presented the repeat size distribution of those rarely-studied dSTR loci by LRS data in a population-scale. Some of those dSTR loci (such as *SAMD12* and *RFC1*) are very dynamic in normal individuals (Figure 2). The distribution of repeat size at other dSTR loci, which are not listed here, were shown in our reference database TRcards (http://www.genemed.tech/trcards/home). Of course, besides the known disease-associated STR loci, the data of all available STR in our dataset are also displayed on our website. For the first time, we presented the repeat size distribution of STR loci with a large scale of LRS data in the general population.

### dSTRs and eSTRs Are Highly Variable

STRs are highly mutable, and thousands of STRs in each individual are different from the reference genome. It is necessary to predict which repeat alterations are likely to be pathological or important. To prioritize potentially important STRs, we are specifically interested in the variability of STRs.

The research found that some dSTRs may be polymorphic and show distinct variation in the general population compared to other STR loci (Mitsuhashi et al., 2021). Besides, expression STRs (eSTRs) and the top fine-mapped eSTRs (FM-eSTRs) were identified as possibly contributing to a range of human phenotypes and being causal (Fotsing et al., 2019). Those eSTRs and FM-eSTRs are a valuable resource for studying the role of STRs in complex traits, and it is worth exploring whether the variability of those eSTRs and FM-eSTRs are the same as dSTRs.

**FIGURE 3:**
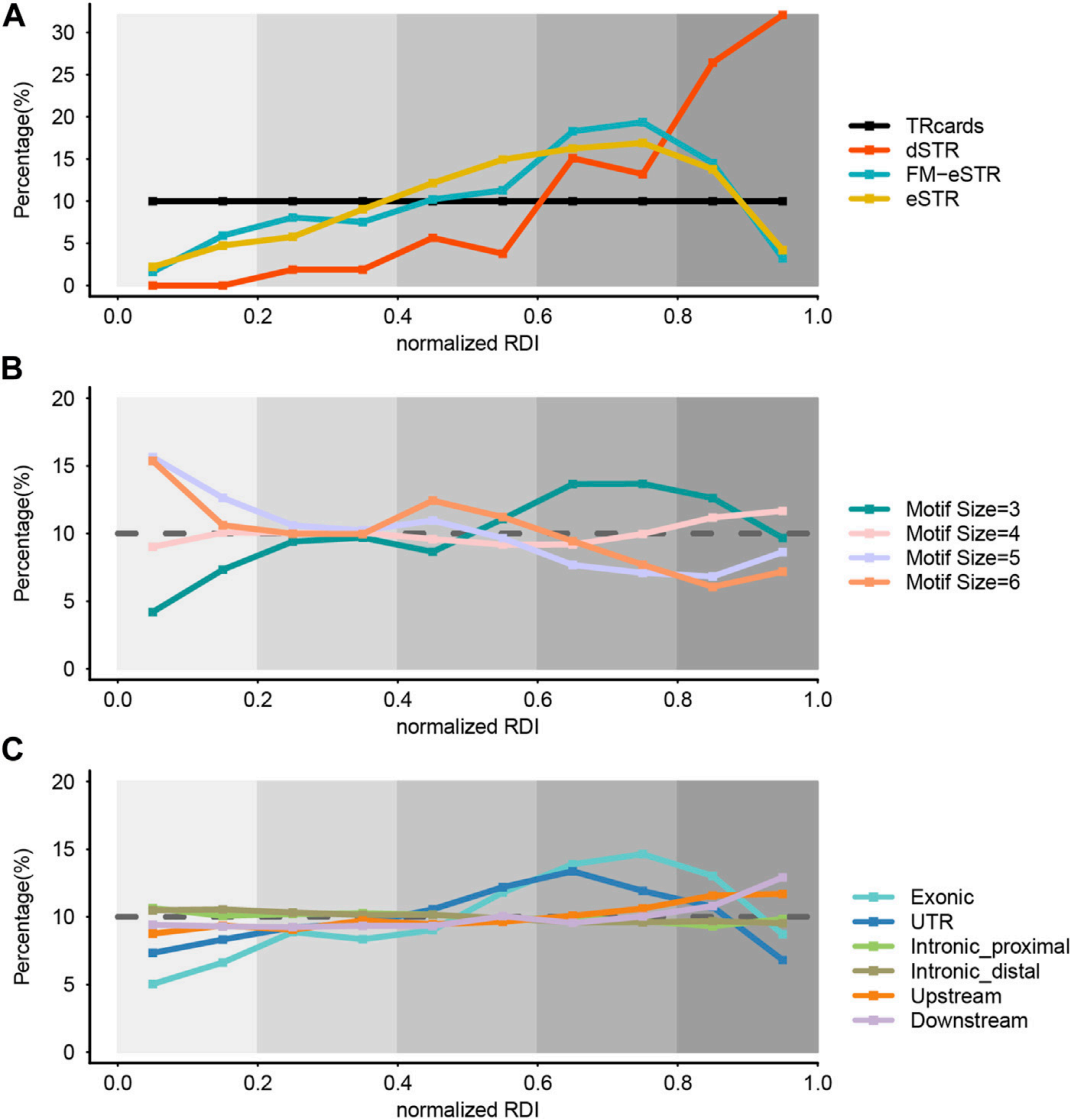
Genome-wide evaluation of STR variability. **(A)** The distribution of STR variability in the dSTR subset, eSTR subset, and FM-eSTR subset. TRcards = our entire STR dataset, dSTR = disease-associated STR, eSTR = expression STRs, FM-eSTR = fine-mapped eSTRs. **(B)** The distribution of STR variability in the different motif size subsets. **(C)** The distribution of STR variability in the different genomic region subsets. In all panels, the *x*-axis gives the normalized RDI value and the *y*-axis gives the percentage of STR loci. Normalized RDI = normalized repeat dynamic index. We refer to the STR with normalized RDI score at 0–0.2, 0.2–0.4, 0.4–0.6, 0.6–0.8, and 0.8–1.0 as very lowly variable (vlSTR), lowly variable STR (lSTR), moderately variable STR (mSTR), highly variable STR (hSTR), and very highly variable STR (vhSTR), respectively. Colors denote different STR subsets. The brown dashed line in **(B)** and **(C)** shows the reference percentage in the entire dataset.

We introduced a repeat dynamic index (RDI) to score the variability for every STR locus. STRs were ranked by the RDI score, and the RDI score of each STR was substituted with a normalized RDI (nRDI) score to address the potential bias. STRs were defined as very lowly variable (vlSTR), lowly variable STR (lSTR), moderately variable STR (mSTR), highly variable STR (hSTR), and very highly variable STR (vhSTR) according their nRDI value. To show STR distribution in a series over the nRDI score, we further divided STRs into ten parts and combined the STRs located in the same part. Our entire STR dataset (TRcards) presented an evenly distributed pattern as a control (Figure 3A). Then, we inspected the distribution of STRs based on the nRDI score in the dSTR subset, eSTR subset, and FM-eSTR subset from our total STR dataset with LRS data. We observed that the proportion of vlSTR and lSTR is small in the dSTR subset, eSTR subset, and FM-eSTR subset, but the proportion of vhSTRs (58.49%) and hSTR (28.30%) is large in the dSTR subset, and the proportion of hSTR is large in the eSTR subset (33.14%) and FM-eSTR subset (37.63%) (Figure 3A). It implies that dSTRs are more common with vhSTR, and both eSTRs and FM-eSTRs are more common with hSTR. This observation is concordant with previous studies that dSTRs are more polymorphic in normal individuals than other repeats (Mitsuhashi et al., 2021). Moreover, our study found that eSTRs associated with gene expression are also more dynamic among the general population than other STR loci. Interestingly, our analysis also proved that eSTRs are less variable than dSTR, which could somehow explain why eSTRs may be not pathogenic as dSTR and just subtly increase or decrease the risk for a trait.

Then, we investigated the effect of motif characteristics on the variability of STR in our STR catalog by dividing it into different subsets from our dataset (TRcards) based on the motif size. We found that the proportion of hSTRs is relatively large and the proportion of vlSTRs is small in the trinucleotide STRs subset, whereas the proportion of vhSTRs is relatively small and the proportion of vlSTR is large in the penta-nucleotide and hexa-nucleotide STRs (Figure 3B). It suggests that the variability of STR decreased with motif length, and the trinucleotide STRs have the highest mutation rates. There is no consensus in the literature regarding the effect of motif characteristics on STR variability.

Next, we stratified the STR dataset based on the genomic features to investigate the effect of the generic region on the variability of STR. We found that STR with different nRDI score is evenly distributed in the intronic region (including distal region and proximal from the exon), upstream region, and downstream region, but the proportion of hSTRs and vhSTRs is relatively large in the exon STR subset and the UTR STR subset, which means that STR is more variable in the coding region and UTR region compared to the intronic region (Figure 3C).

Moreover, we also took a closer examination of the STR variability in TRcards, dSTR subset, eSTR subset, and FM-eSTR subset with different motif sizes and different genomic regions. It showed a similar distribution pattern as the above results (Supplementary Figure S5, S6).

### vhSTRs and hSTRs Are Enriched in the Brain

Because STRs that are disease-causing and functionally impactful tend to be highly variable in the general population, meaning that those highly variable STRs (vhSTRs and hSTRs) may be more correlated with human genetic diseases or complex traits. We next sought to characterize the properties of hvSTRs and hSTRs that might provide insights into their biological function. To delineate the possible functional roles of those hvSTRs and hSTRs, we investigated the tissue-specific expression of those STRs related genes.

**FIGURE 4:**
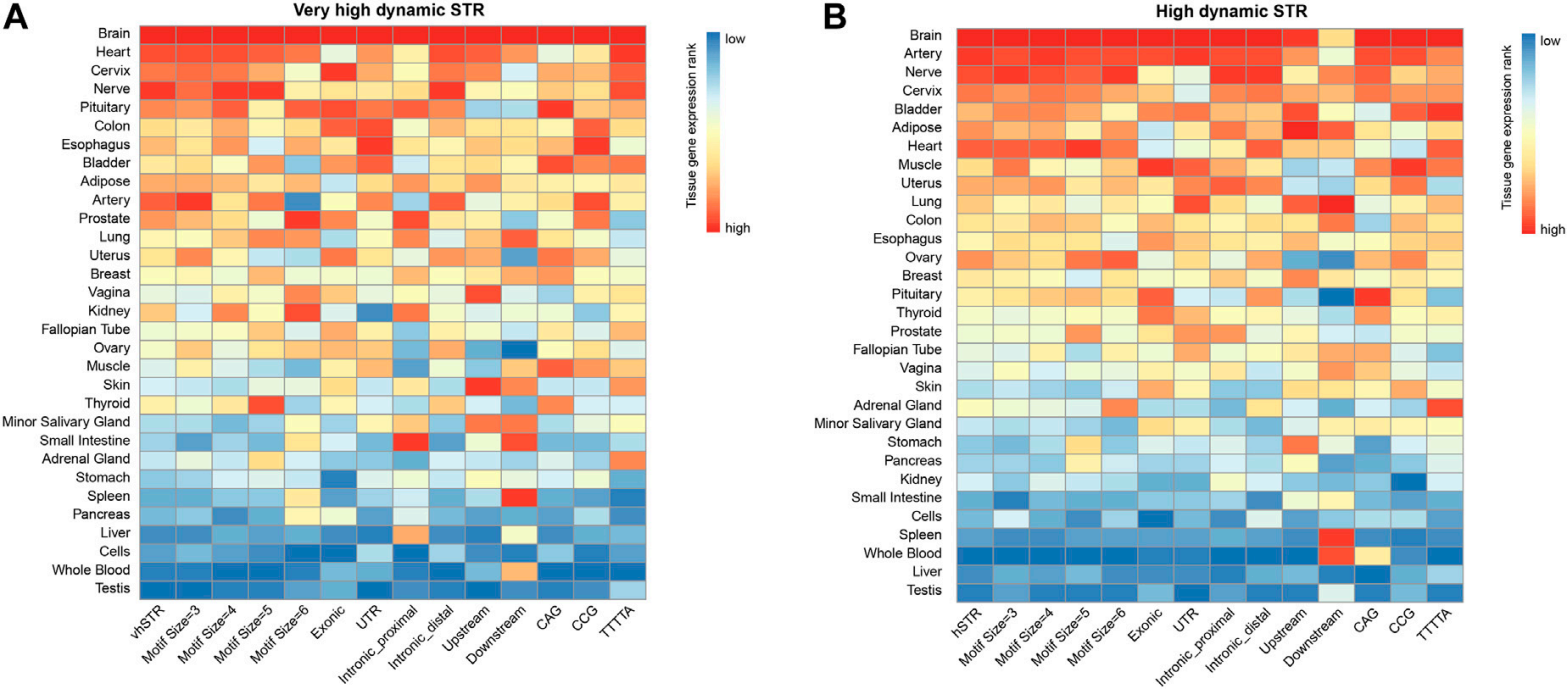
Tissue-specific expression profiles of vhSTRs and hSTRs. **(A)** Tissue-specific expression pattern of vhSTRs. **(B)** Tissue-specific expression pattern of hSTRs. Heatmaps show the expression patterns of different STRs subset across different tissues based on the normalized expression level. vhSTRs = very highly variable STRs, hSTRs = highly variable STRs. The rows represent the entire dataset of vhSTR or hSTR and their subsets stratified by different motif sizes, genomic regions, and repeat units. The columns represent the tissues.

Herein, we calculated the preferential expression of tissues of hvSTRs and hSTRs related genes and systematically tested the enrichment of preferential expression tissues using expression data derived from the Genotype-Tissue Expression (GTEx) database. Strikingly, we found that those vhSTRs and hSTR related genes were more likely to be expressed in brain tissue than non-brain tissues (Figures 4A,B). In addition to brain tissue, other tissues specifically expressed are the heart, artery, cervix, and nerve. We stratified vhSTRs and hSTR based on the genomic features, motif sizes, and dSTR repeat units and observed that the expression patterns of those STRs subsets were very compatible and most enriched in the brain tissue (Figure 4, Supplementary Figure S7, S8, S9). These results provide novel evidence that vhSTRs and hSTRs are likely involved in brain specific gene regulation.

### vhSTRs and hSTRs Are Involved in Synaptic Function

**FIGURE 5:**
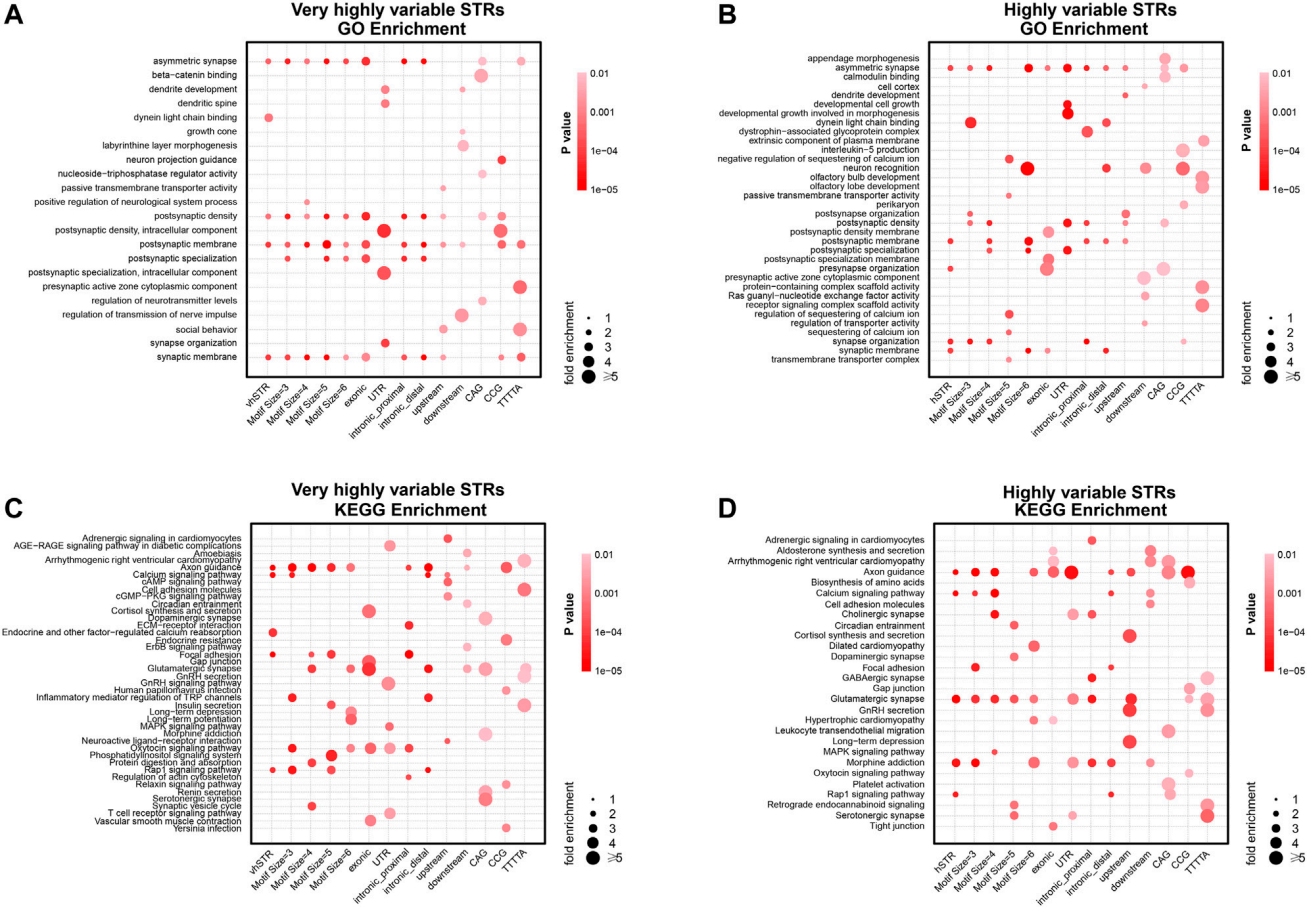
Enrichment pathways of vhSTRs and hSTRs. **(A)** Significant GO terms of vhSTR. **(B)** Significant GO terms of hSTR. **(C)** Significant KEGG pathways of vhSTR. **(D)** Significant KEGG pathways of hSTR. vhSTRs = very highly variable STRs, hSTRs = highly variable STRs. GO = Gene Ontology, KEGG = Kyoto Encyclopedia of Genes and Genomes. In all panels, the rows represent the entire dataset of vhSTR or hSTR and their subset stratified by different motif sizes, genomic regions, and eSTR repeat units. The columns represent the GO terms or KEGG pathway. Fisher’s exact test was used to calculate the *p*-value for each tissue.

To elucidate the biological pathways of hvSTRs and hSTR, we investigated their relevance to pathways using the pathway enrichment analysis. The vhSTRs related genes are enriched in multiple aspects of synaptic function by Gene Ontology (GO) analysis, notably asymmetric synapse, postsynaptic density, postsynaptic membrane, and synaptic membrane (*p*-value＜0.01). According to the Kyoto Encyclopedia of Genes and Genomes (KEGG) pathway analysis, those vhSTRs related genes were predominantly involved in the axon guidance, calcium signaling pathway, endocrine and other factor-regulated calcium reabsorption, focal adhesion, and Rap1 signaling pathway (*p*-value＜0.01) (Figures 5A,C). We also took an enrichment analysis of different vhSTR subsets, stratifying the vhSTR gene set according to different motif sizes, genomic regions, and dSTR repeat units. The GO categories of most of these vhSTR subsets are similar to the entire vhSTR gene set, in addition to the vhSTR subset in the UTR region, downstream region, and CAG unit, whose enrichment categories include dendrite development, dendrite spine, postsynaptic density, postsynaptic specialization, and synaptic organization (*p*-value＜0.01). For the KEGG analysis of different vhSTR subsets, most of the subsets are not consistent with the total set. However, the enriched pathways are all mainly related to neuron function, such as the glutamatergic synaptic and synaptic vesicle cycle (*p*-value＜0.01) (Figures 5A,C).

For the hSTR related genes, the significant GO categories included asymmetric synapse, postsynaptic membrane, presynaptic organization, synapse organization, and synaptic membrane, all of which are synaptic functions (*p*-value＜0.01). The hSTRs were significantly enriched in the KEGG pathways, including axon guidance, glutamine synapse, and calcium signaling pathway (*p*-value＜0.01) (Figures 5B,D). Different hSTR subsets, stratified according to different motif sizes, genomic regions, and dSTR repeat units, were also performed with GO and KEGG analysis. The significant GO categories of the hSTR subset are not consistent with the entire hSTR set, including dynein light chain binding, neuron recognition, and development cell growth developmental growth in morphogenesis (*p*-value＜0.01). We also took a KEGG analysis of different hSTR subsets and found that the enrichment pathways of most of these hSTR subsets are similar to the entire hSTR gene set (*p*-value＜0.01) (Figures 5B,D).

Together, these results support that hvSTRs and hSTR may act as important drivers of neurodevelopment disease and neurodegenerative disorders.

### TRcards: A Reference Database of Normal Repeat Range for STRs

**FIGURE 6:**
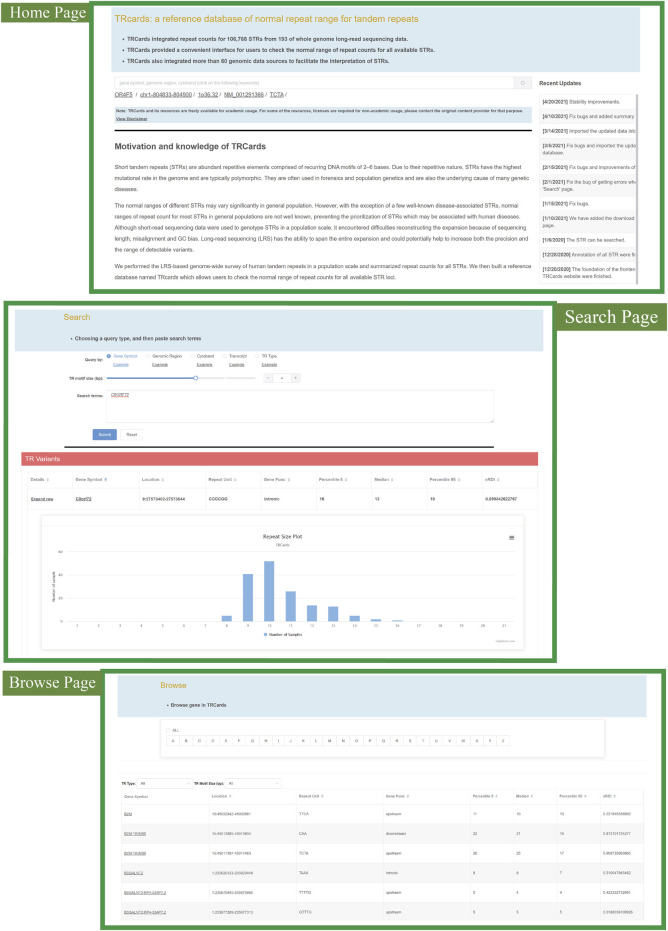
Snapshot of the TRcards web interface (http://www.genemed.tech/trcards/home). “Home page” shows the introduction and motivation of TRcards. There are approaches to access specific STRs in the “Search page” and “Browse page” through different input query types. CACCC repeat in FAM41C gene is illustrated as an example to show the information for each STR locus, including the chromosome, the starting position of the repeat, the end position of the repeat, the 5 and 95 percentile of the repeat counts, nRDI value, and the plot of repeat size distribution.

We developed a reference database of normal repeat range for all STR loci, named TRcards (http://www.genemed.tech/trcards/home), which integrated repeat counts for all available STRs with 193 ONT LRS datasets (Figure 6). TRcards features a user-friendly query interface and provides a comprehensive overview of STRs and their annotation information. More than 60 popular genomic data sources were also integrated into TRcards to provide users with comprehensive information regarding STRs and genes. The website provides a series of graphical interfaces to search for STR loci with specific biological properties and obtain summary statistics. The search function is the main tool to quickly access detailed information regarding STRs and can be found on the home page (Figure 6). The search automatically recognizes a variety of key terms, such as gene symbol, genomic region, cytoband, transcript, or TR type. Moreover, several examples of input query formats are available by clicking the “example” link with the corresponding examples occurring in the input box (Figure 6). For every STR locus, TRcards show information about STR, including the chromosome, the starting position of the repeat, the end position of the repeat, the 5 and 95 percentile of the repeat counts, nRDI value, and the plot of repeat size distribution with 193 available LRS data (Figure 6). Users can simply extract normal repeat range information of STR loci.

## Discussion

Short tandem repeats are abundant repetitive elements throughout the human genome and show a clear implication in human disease and complex traits. Short-read sequencing has been used to profile STRs and could provide a good estimate, but genotyping STRs from short-read sequencing has proven challenging because short reads do not span entire repeats and induce mapping bias. Long-read sequencing provides advantages when the repeat length is more than 150 bp and could address the challenges and offer a good solution to genome-wide STR analysis (Amarasinghe et al., 2020; Logsdon et al., 2020). To our knowledge, the current study represents the largest analysis of human whole-genome long-read sequencing data to detect STRs and greatly expands the growing information on STR variations.

Due to their repetitive nature, STRs are typically highly mutable in the human genome. Thousands of STRs in each individual are different from the reference genome, which challenges predicting which STR could cause disease (Liu et al., 2020). In addition to STRs linked to human genetic disease (dSTR), STRs associated with the expression of nearby genes (eSTRs) are also reported to contribute to a range of human phenotypes (Fotsing et al., 2019). We introduced a repeat dynamic index (RDI) to score the variability for every STR locus and genome-wide evaluate the variability of STRs using our LRS-based STR dataset. Strikingly, our analysis found that dSTRs and eSTRs are highly variable among the general population than other STR loci. This observation is concordant with previous studies that dSTRs are polymorphic in the general population. Moreover, our study also implied that eSTRs are less variable than dSTR, suggesting that eSTRs may be less pathogenic than dSTR and only subtly increase or decrease the risk for human phenotypes.

Notably, our analysis found that vhSTRs and hSTRs are enriched in the brain, and the most enriched pathways were predominantly involved in the synaptic function. To date, STR expansions are linked to at least 50 known disorders, and many of these conditions affect the nervous system, including neurodevelopment disorders and neurodegenerative disorders (Hannan, 2018; Depienne and Mandel, 2021). The hvSTR and hSTR catalogs, highlighted by our study, provide a valuable resource for studying the role of STRs in human disease and complex traits, which helps identify novel disease-causing STR candidates. Variability in such tandem repeats may contribute to the missing heritability of many common disorders (Hannan 2010). Because only healthy individuals are included in our study, we did not investigate the role of those hvSTRs and hSTRs in human disease at this moment, and additional work in the future to directly investigate associations between those vhSTR and hSTRs and phenotypes may reveal a role for STR variation in human phenotypes.

In addition, the normal ranges of STRs vary significantly in the general population, and the knowledge of the normal repeat ranges of STRs is critically important to determine the pathogenicity of observed repeats in known STRs or to discover novel disease-relevant repeat expansions (Liu et al., 2020). In order to facilitate future studies, TRcards, a reference database of repeat counts for all STRs with the LRS dataset, were built in our study. TRcards is a user-friendly, open-access web-based interface to browse and search the STRs and can be very useful to pinpoint abnormal repeat counts for human disease studies. A recent study developed RepeatHMM-DB based on 21 available long-read sequencing datasets, which, as proposed, could be useful to facilitate prioritization and identification of disease-relevant STRs from whole-genome long-read sequencing data. Nevertheless, RepeatHMM-DB did not present the normal repeat ranges of STRs on the website (Liu Q. 2020).

However, there are several limitations. First, our study only investigated the STRs with the common motif sizes (3-6 bp); we will supplement STR with other motif sizes, including variable number of tandem repeats in the future. Second, we only included STRs located within the 10 kb region of the nearby gene, which are more associated with the expression of nearby genes. Other STRs located beyond this defined region will be overcome. Third, currently, the STR loci in TRcards are coordinated on the GRCh37/hg19, and we will also provide STR information for the GRCh38/hg38 coordinate in the future. Finally, despite strong evidence showing that the hvSTRs and hSTRs are important, future work is needed to directly evaluate the impact of those vhSTR and hSTRs in trait.

## Conclusion

In conclusion, this study reports the large-scale analysis of human STR variation by LRS and offers a reference STR database based on the LRS dataset. We profiled the genome-wide landscape of STR and highlighted the highly variable STRs catalog, providing a valuable resource for studying the role of STRs in human disease and complex traits.

## Data Availability

According to national legislation/guidelines, specifically the Administrative Regulations of the People’s Republic of China on Human Genetic Resources (http://www.gov.cn/zhengce/content/2019-06/10/content_5398829.htm, http://english.www.gov.cn/policies/latest_releases/2019/06/10/content_281476708945462.htm), no additional raw data are available at this time. Data of this project can be accessed after an approval application to the China National Genebank (CNGB, https://db.cngb.org/cnsa/). Please refer to https://db.cngb.org/ or email: CNGBdb@cngb.org for detailed application guidance. The accession code CNP0002362 should be included in the application.
