# Supplementary figures and images for: Profiling the Genome-Wide Landscape of Short Tandem Repeats by Long-Read Sequencing
Source: Front Genet. 2022 May 5;13:810595. doi: 10.3389/fgene.2022.810595 (PMC9117641; doi:10.3389/fgene.2022.810595)

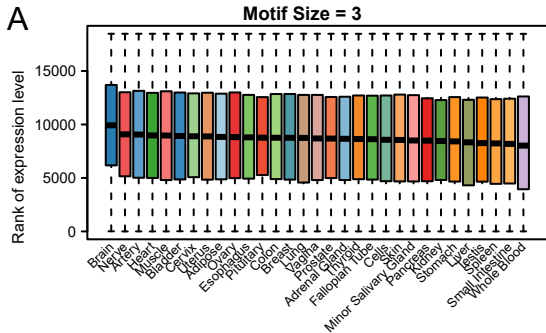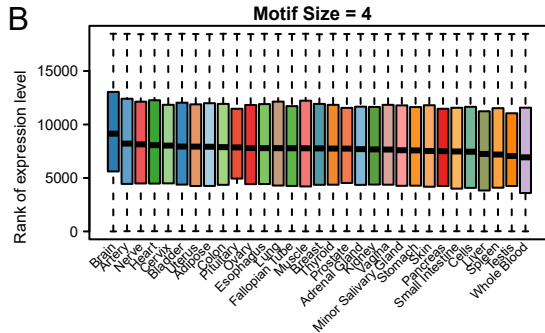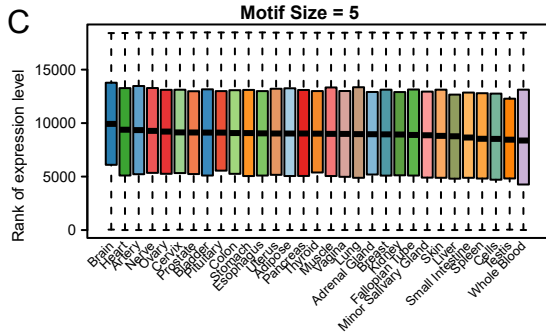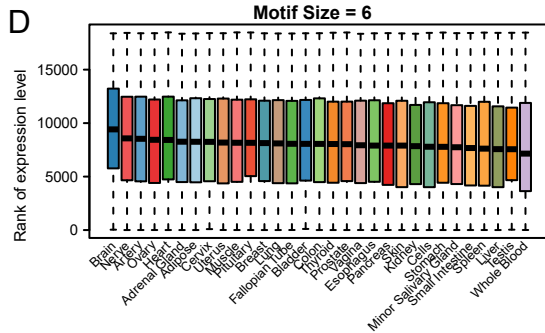

Supplement: Supplementary file 1 [file DataSheet7.PDF]

A

ONT

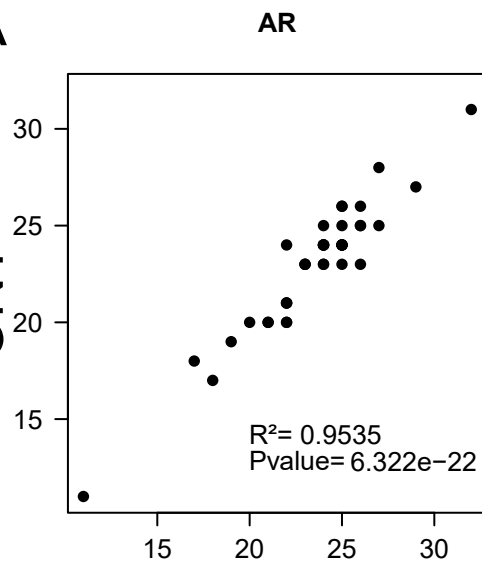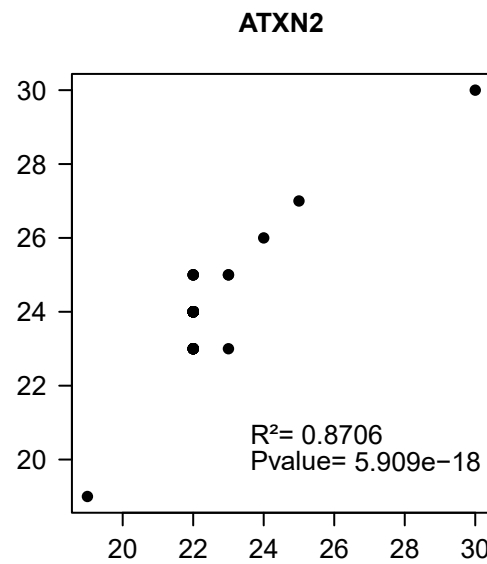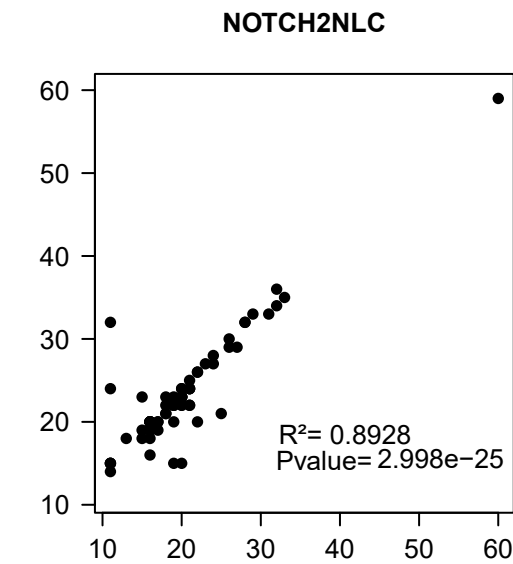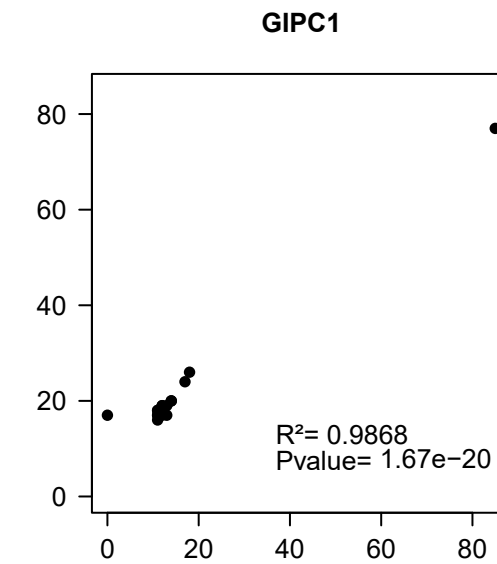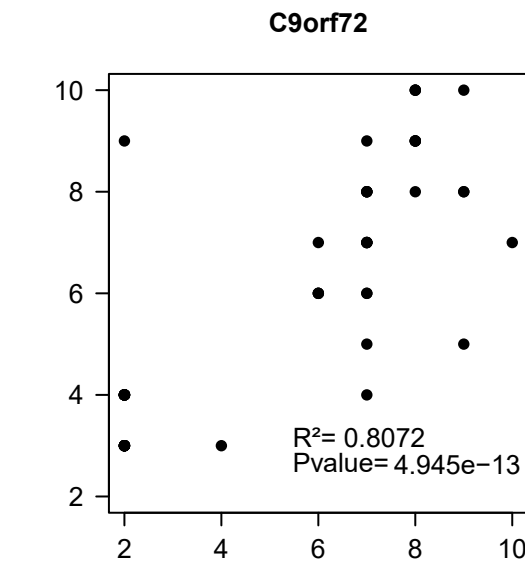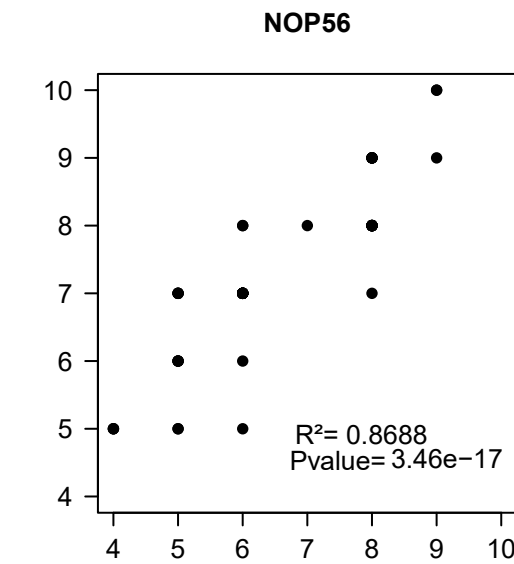

B

Difference

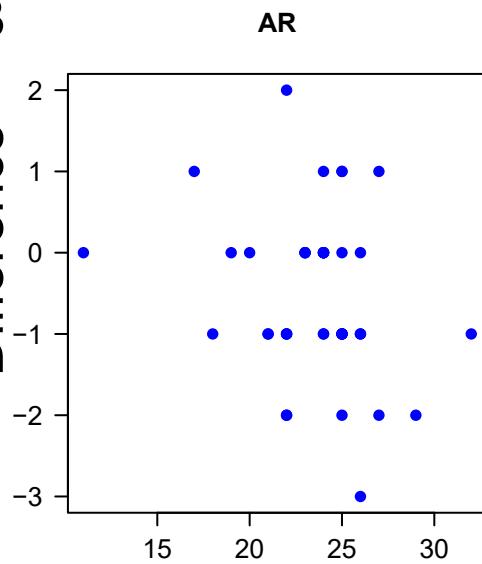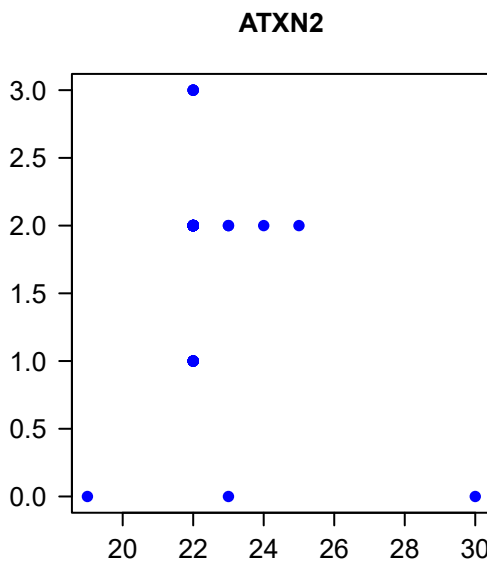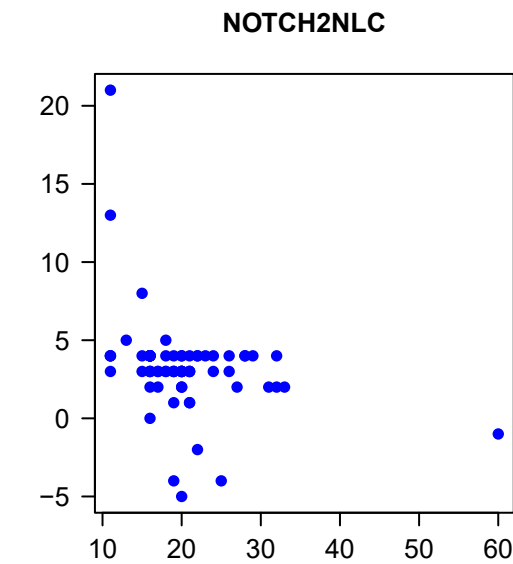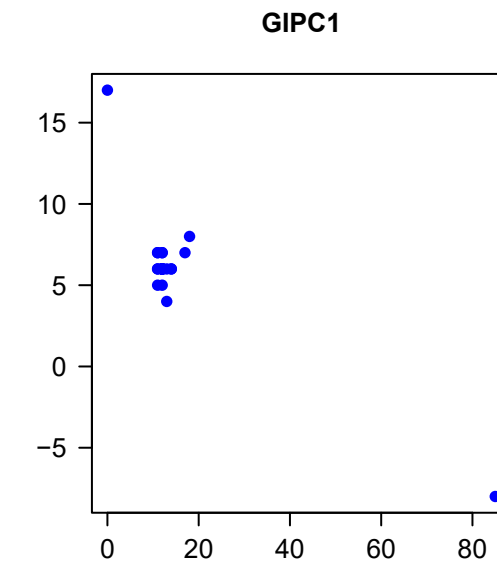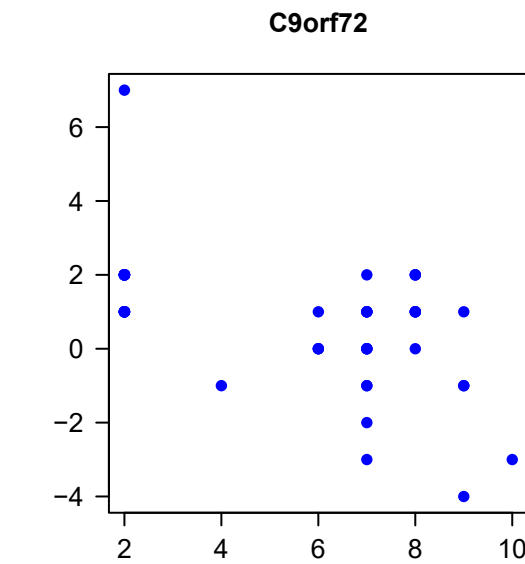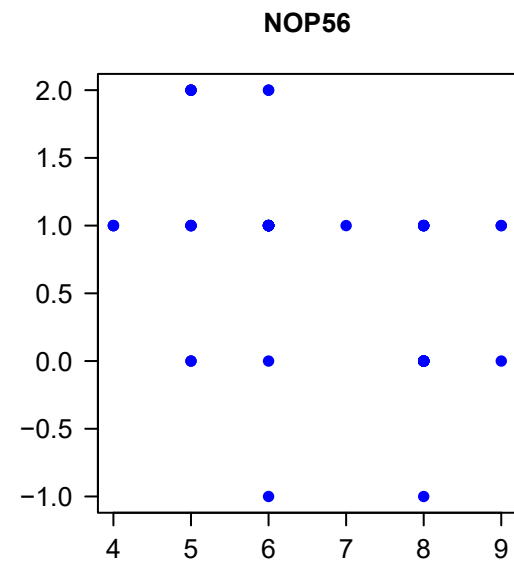

Capillary electrophoresis

Supplement: Supplementary file 4 [file DataSheet4.PDF]

— TRcards — dSTR — FM-eSTR — eSTR

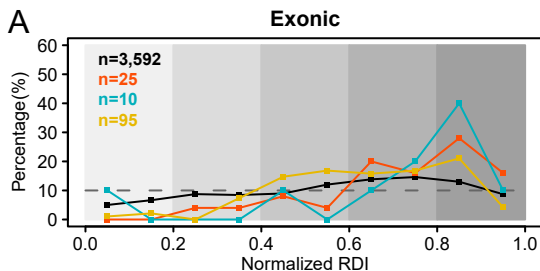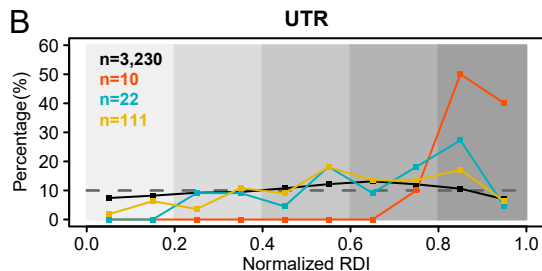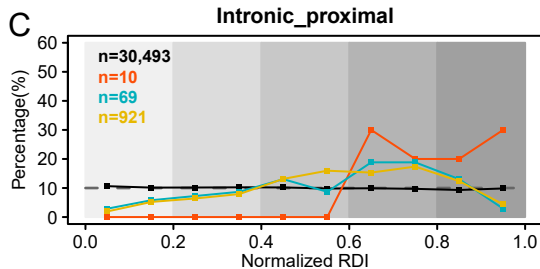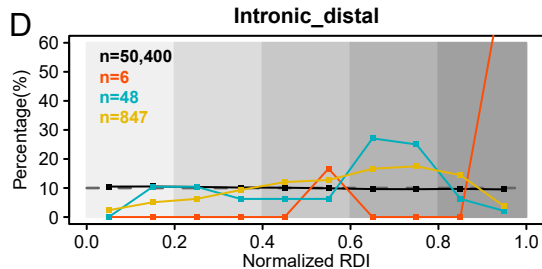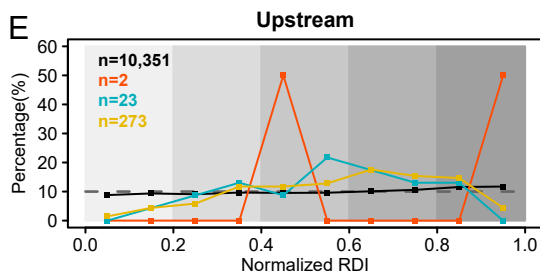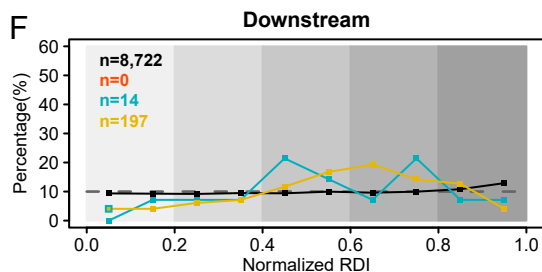

Supplement: Supplementary file 5 [file DataSheet6.PDF]

A

CAG

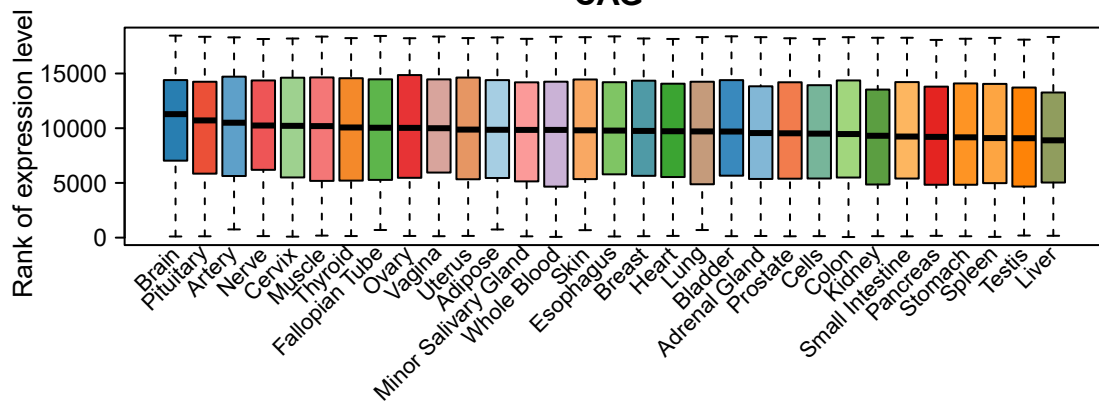

B

CCG

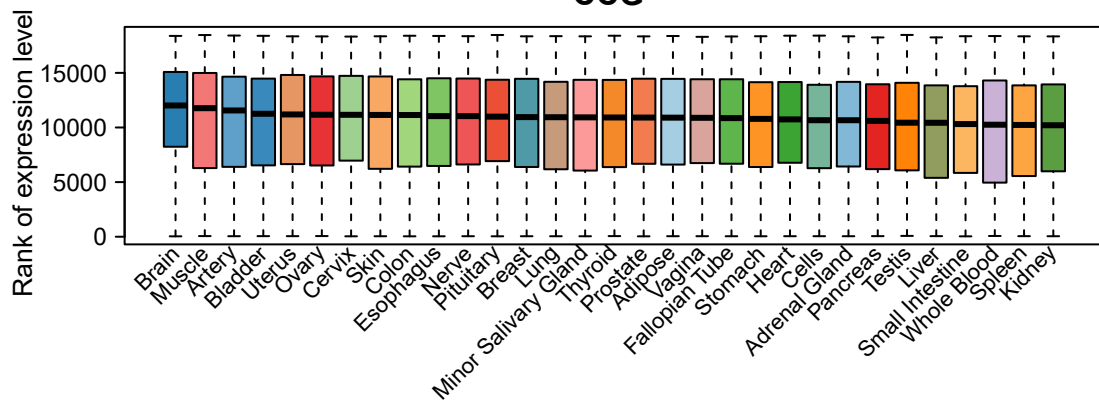

C

TTTTA

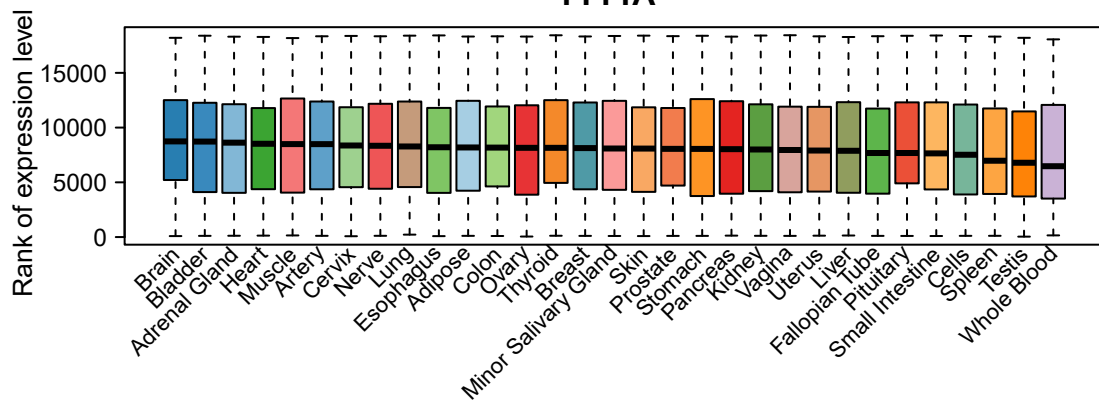

Supplement: Supplementary file 8 [file DataSheet9.PDF]

**A** HG002

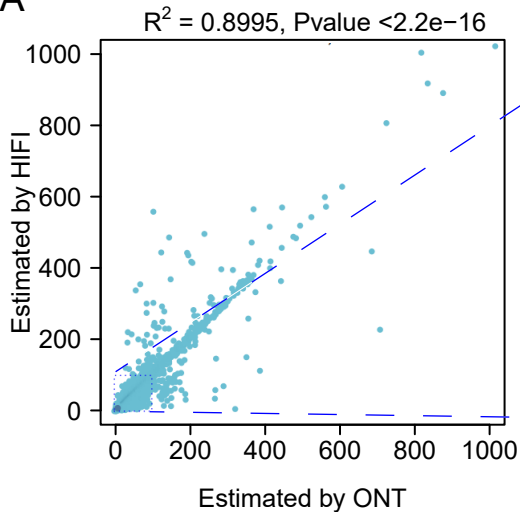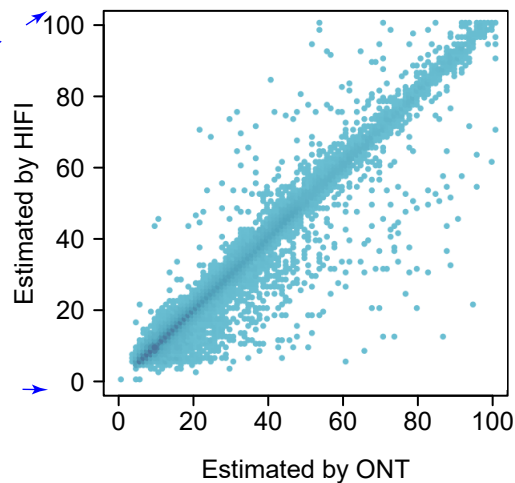

**B** LNT00178

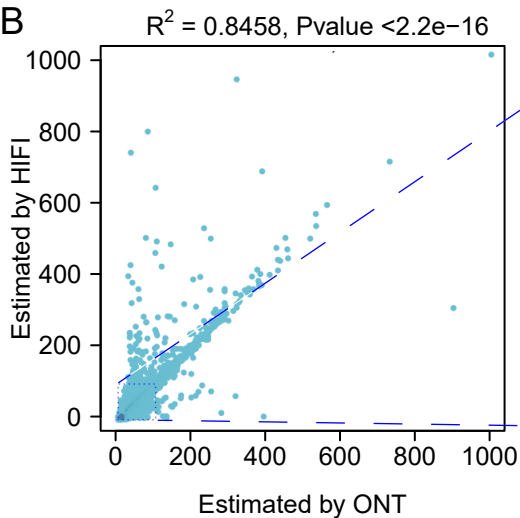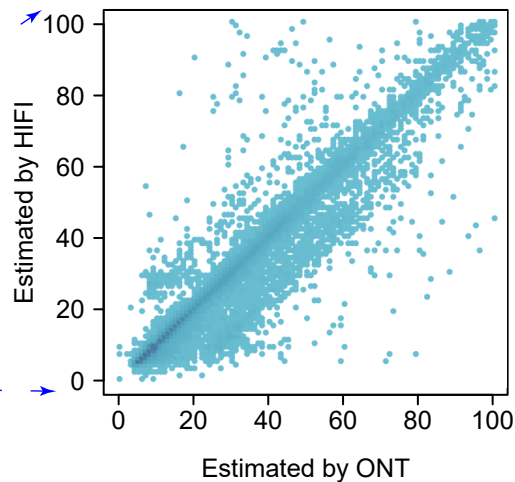

Supplement: Supplementary file 10 [file DataSheet3.PDF]

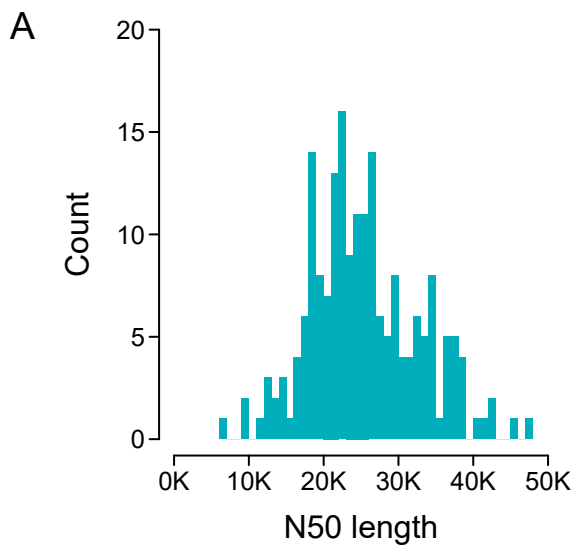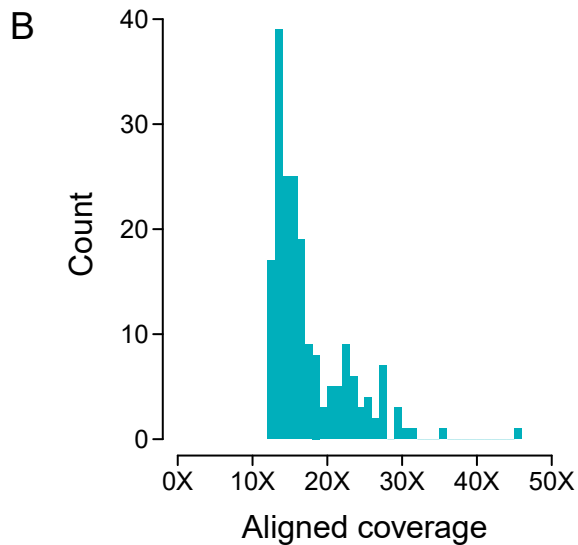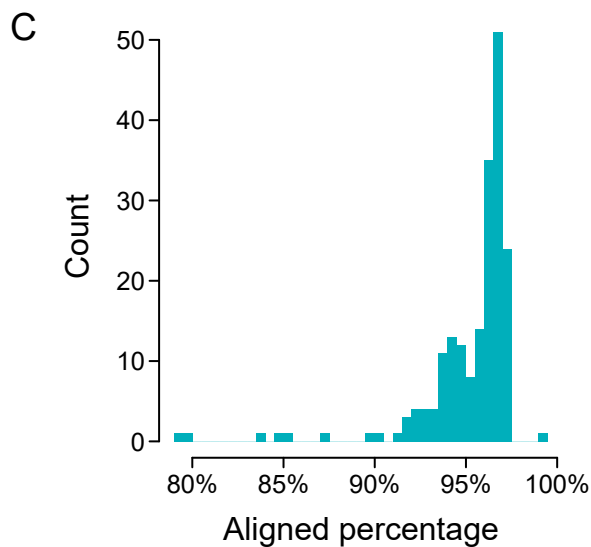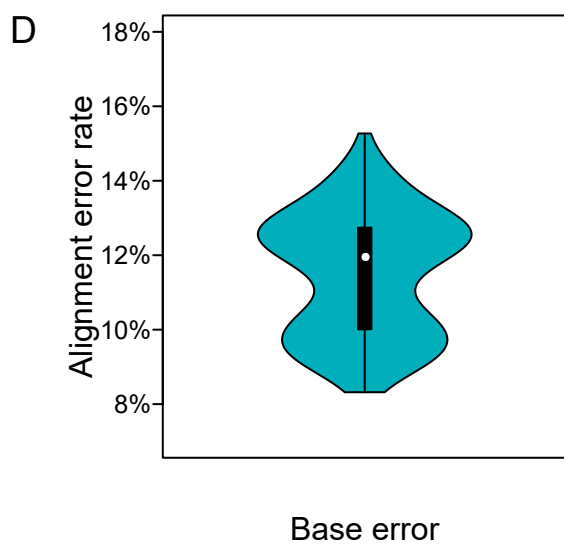

Supplement: Supplementary file 12 [file DataSheet1.PDF]

—■— TRcards —■— dSTR —■— FM-eSTR —■— eSTR

**A**

**Motif Size = 3**

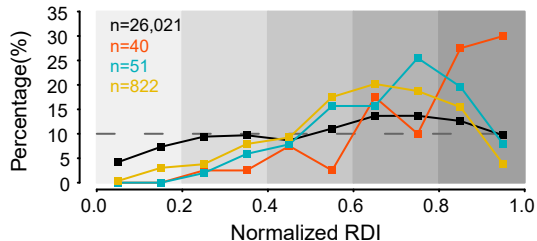

**B**

**Motif Size = 4**

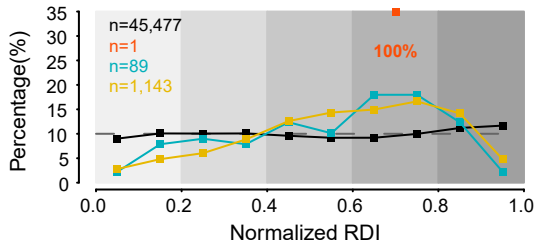

**C**

**Motif Size = 5**

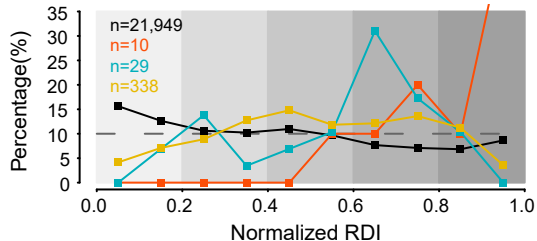

**D**

**Motif Size = 6**

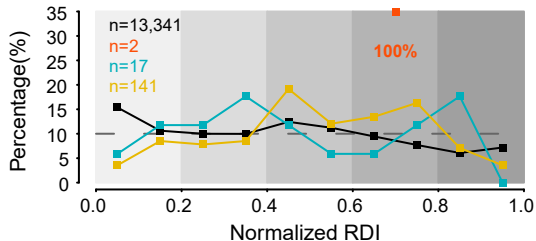

Supplement: Supplementary file 13 [file DataSheet5.PDF]

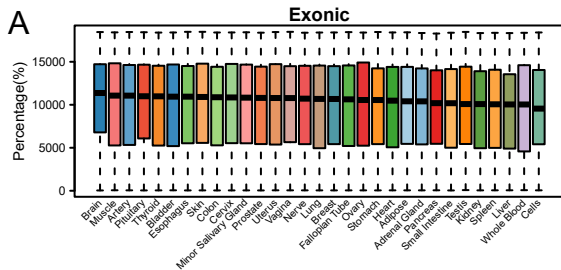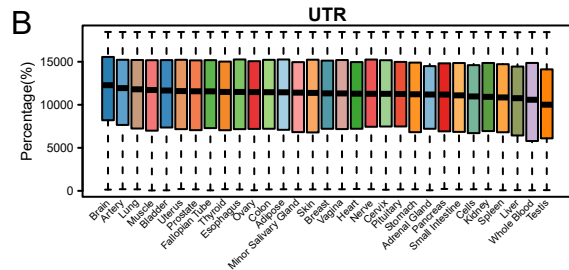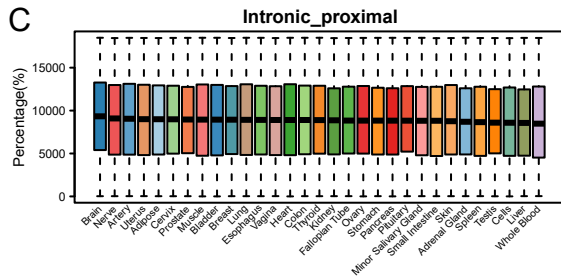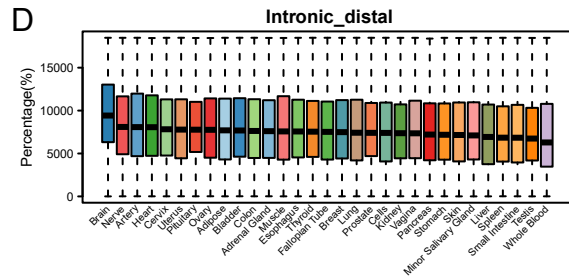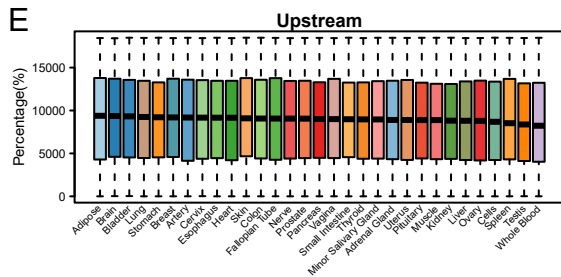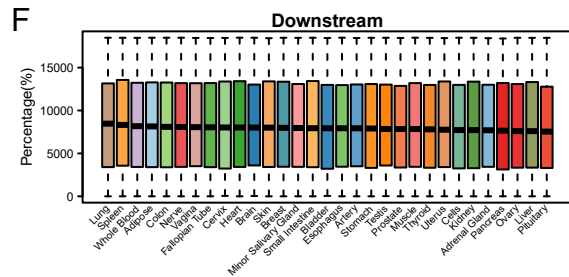

Supplement: Supplementary file 16 [file DataSheet8.PDF]
