# Supplementary material for: Profiling the Genome-Wide Landscape of Short Tandem Repeats by Long-Read Sequencing
Source: Front Genet. 2022 May 5;13:810595. doi: 10.3389/fgene.2022.810595 (PMC9117641; doi:10.3389/fgene.2022.810595)
Supplement: Supplementary file 2 [file DataSheet2.PDF]

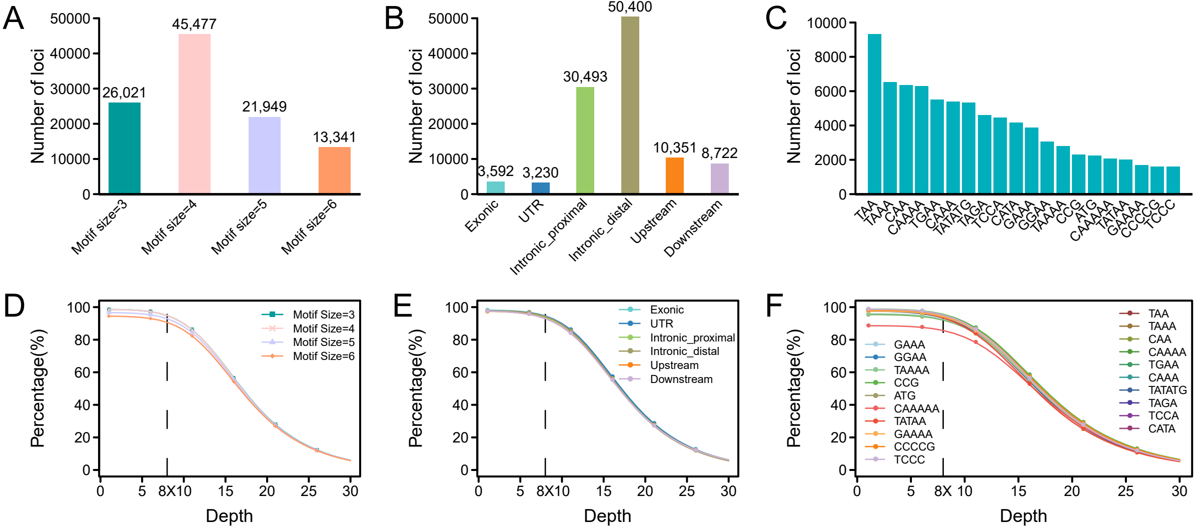

A
Number of loci
26,021
45,477
21,949
13,341
Motif size=3
Motif size=4
Motif size=5
Motif size=6
B
Number of loci
3,592
3,230
30,493
50,400
10,351
8,722
Exonic
UTR
Intronic_proximal
Intronic_distal
Upstream
Downstream
C
Number of loci
TAA
TAAA
CAA
CAAAA
TGAA
CAAA
TATATG
TAGA
TCCA
CATA
GAAA
GGAA
TAAAA
CCG
ATG
CAAAAA
TATAA
GAAAA
CCCCG
TCCC
D
Percentage(%)
Motif Size=3
Motif Size=4
Motif Size=5
Motif Size=6
8X
Depth
E
Percentage(%)
Exonic
UTR
Intronic_proximal
Intronic_distal
Upstream
Downstream
8X
Depth
F
Percentage(%)
GAAA
GGAA
TAAAA
CCG
ATG
CAAAAA
TATAA
GAAAA
CCCCG
TCCC
TAA
TAAA
CAA
CAAAA
TGAA
CAAA
TATATG
TAGA
TCCA
CATA
8X
Depth
